# Evolutionary Modifications Are Moderate in the Astroglial System of Actinopterygii as Revealed by GFAP Immunohistochemistry

**DOI:** 10.3389/fnana.2021.698459

**Published:** 2021-06-29

**Authors:** Mihály Kálmán, Vanessza Matuz, Olivér M. Sebők, Dávid Lőrincz

**Affiliations:** ^1^Department of Anatomy, Histology, and Embryology, Semmelweis University, Budapest, Hungary; ^2^Department of Zoology, University of Veterinary Medicine, Budapest, Hungary

**Keywords:** astrocytes, cerebellum, eversion, radial glia, telencephalon, tectum

## Abstract

The present paper is the first comparative study on the astroglia of several actinopterygian species at different phylogenetical positions, teleosts (16 species), and non-teleosts (3 species), based on the immunohistochemical staining of GFAP (glial fibrillary acidic protein), the characteristic cytoskeletal intermediary filament protein, and immunohistochemical marker of astroglia. The question was, how the astroglial architecture reflexes the high diversity of this largest vertebrate group. The actinopterygian telencephalon has a so-called ‘eversive’ development in contrast to the ‘evagination’ found in sarcopterygii (including tetrapods). Several brain parts either have no equivalents in tetrapod vertebrates (e.g., torus longitudinalis, lobus inferior, lobus nervi vagi), or have rather different shapes (e.g., the cerebellum). GFAP was visualized applying DAKO polyclonal anti-GFAP serum. The study was focused mainly on the telencephalon (eversion), tectum (visual orientation), and cerebellum (motor coordination) where the evolutionary changes were most expected, but the other areas were also investigated. The predominant astroglial elements were tanycytes (long, thin, fiber-like cells). In the teleost telencephala a ‘fan-shape’ re-arrangement of radial glia reflects the eversion. In bichir, starlet, and gar, in which the eversion is less pronounced, the ‘fan-shape’ re-arrangement did not form. In the tectum the radial glial processes were immunostained, but in Ostariophysi and Euteleostei it did not extend into their deep segments. In the cerebellum Bergmann-like glia was found in each group, including non-teleosts, except for Cyprinidae. The vagal lobe was uniquely enlarged and layered in Cyprininae, and had a corresponding layered astroglial system, which left almost free of GFAP the zones of sensory and motor neurons. In conclusion, despite the diversity and evolutionary alterations of Actinopterygii brains, the diversity of the astroglial architecture is moderate. In contrast to Chondrichthyes and Amniotes; in Actinopterygii true astrocytes (stellate-shaped extraependymal cells) did not appear during evolution, and the expansion of GFAP-free areas was limited.

## Introduction

**FIGURE 1:**
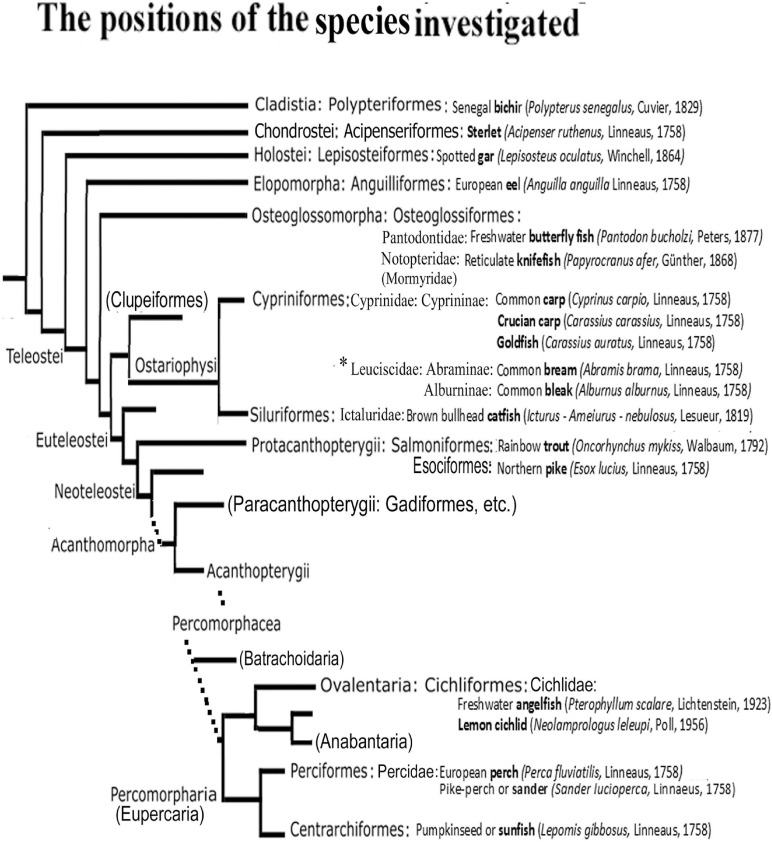
The positions of the species studied in the phylogenetic tree. After Betancur-R et al. (2013, 2017), simplified. Only the taxa relevant for understanding the study are introduced. The short names in bold are used throughout the text and Legends. Families and subfamilies are only given when more than one are represented of the same ordo in the study including the Appendix 1. Ordos named in bracket have no representatives investigated here, but do have in the Appendix 1, or help general orientation. Dotted lines: the several intermediate branchings are not demonstrated. The main differences from other taxonomies are discussed at the end of Discussion. ^∗^A recent study (Schönhut et al., 2018) separated Leuciscinae from Cyprinidae as Leuciscidae.

The present paper is the first comparative study on the astroglia of several actinopterygian species at different phylogenetical positions (Figure 1) based on the immunohistochemical staining of GFAP (glial fibrillary acidic protein, Eng et al., 1971), the characteristic cytoskeletal intermediate filament protein, and immunohistochemical marker of astroglia (Bignami et al., 1980).

Teleostei, the largest group of Actinopterygii, and even Chordata, is a relatively new and efflorescent group of high diversity (for the diversity of their brains see, e.g., Nieuwenhuys and Meek, 1997). Until now GFAP studies usually focused only single species, mainly cyprinids, and the results were extended to all the Teleostei, or even Actinopterygii, disregarding the possible interfamily differences.

The question is, how the astroglial architecture reflexes the evolutionary diversity of Actinopterygii. In Chondrichthyes and Amniotes our former studies found considerable diversity in the astroglial architecture, and a relative withdraw of GFAP immunopositivity during evolution (Kálmán, 2002, 2009; Ari and Kálmán, 2008; Lőrincz and Kálmán, 2020). The present study completes the former ones extending our investigations over the Actinopterygii, and therefore helps to understand the role of GFAP in brain evolution.

**FIGURE 2:**
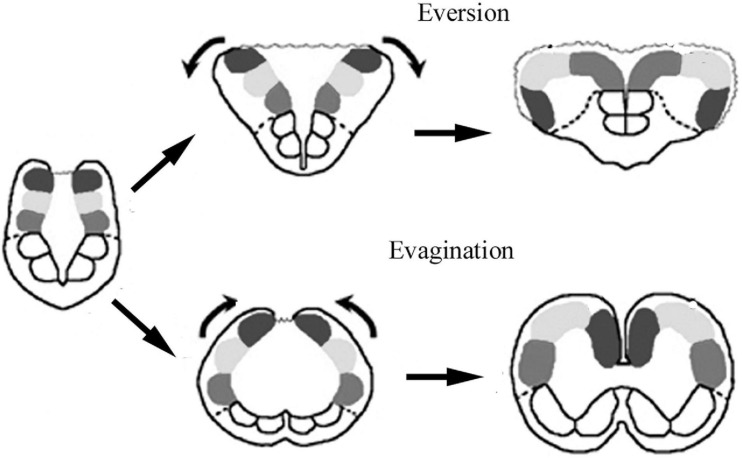
Sketch of brain evagination characteristic of Sarcopterygii-Tetrapoda, and eversion characteristic of Actinopterygii. Solid spots: pallial subdivisions, empty spots: subpallium, dotted line: their border, wavy line: the thin wall of ventricle.

The evolution of Actinopterygii followed a separate course from that of the tetrapods (the sarcopterygian clad). The telencephalon has a so-called ‘eversive’ development in contrast to the ‘inversive’ development (‘evagination’) found in tetrapods (Figure 2), and shows an increasing complexity during evolution (Nieuwenhuys, 1962, 1982; for recent reviews Northcutt, 2008; Nieuwenhuys, 2009, 2011a). which also demonstrates the homologies the areas of these two brain types. Several brain parts either have no equivalents in other vertebrates (e.g., torus longitudinalis, lobus inferior, lobus nervi vagi), or have rather different shapes (e.g., the cerebellum) (Ariens-Kappers et al., 1960; Nieuwenhuys and Meek, 1997). Comparison of basal and advanced teleosts revealed a shift of brain mass during evolution toward these brain parts (Ridet and Bauchot, 1990; Cerda-Reverter et al., 2008).

The present study continues our previous studies on ray-finned fishes (common carp, *Cyprinus carpio*, Kálmán, 1993, 1998; goldfish, *Carassius auratus*, Kálmán and Ajtai, 2000, sterlet, *Acipenser ruthenus*, Kálmán and Ari, 2002). Now representatives of non-teleost groups (Cladistia – also named Brachiopterygii -, Chondrostei, Holostei) were investigated as well as those of basal teleost groups (Osteoglossiformes, Anguilliformes), and those of Ostariophysi (Cypriniformes, Siluriformes) and Euteleostei (Salmoniformes, Cichliformes, Centrarchiformes, Perciformes). The species investigated represented different lifestyles, and inhabited different environments.

The study has been focused mainly on the telencephalon (eversion), tectum (visual orientation), and cerebellum (motor coordination). Presentation of every brain area of every species studied would extend beyond the limits of this paper, therefore, only typical details are shown, and similar areas of different species are demonstrated with one representative figure.

In the present paper, the term ‘radial glia’ is applied to glial processes oriented from the ventricle to the meningeal surface, whereas ‘tanycyte’ (Horstmann, 1954) may refer to any long, fiber-like cells, independent of their origin and orientation. The term ‘astrocyte’ is preserved for the stellate-shaped cells, whereas the term ‘astroglia’ comprises all these and other related glial structures, which can express GFAP (Mugnaini, 1986). Throughout the text the short English names (expressed in bold letters in Figure 1) of fishes are used, which are probably more familiar and readable. The complete specifications are found in Figure 1. The names of species used in the other studies cited are only given in the text where it seemed to be essential. All these names are listed in an ‘Appendix 1’ following the Discussion.

## Materials and Methods

### Animals

The species used in the study and their phylogenetical positions are shown in Figure 1. Tropical fishes were purchased in pet shops. Breeders provided eel (Tamás Müller, Szent István University), trout (Hegedüs Trout Farm at Visegrad), sander (Robert Hegedüs farm, Győr), perch (Bence Frisócky, Ekocenter Tisza-Lake). Sunfish, crucian carp, bleak, bream were presented by local fishermen in living condition. We studied at least 2 animals of each species.

The experiments were performed in accordance with the Committee on the Care and Use of Laboratory Animals of the Council on Animal Care at the Semmelweis University of Budapest, Hungary (22.1/3491/003/2008), the permission of Hungarian authorities (KA-1928, dated from May 31, 1916) and the European Union Directive (EU Directive 2010/63/EU).

### Fixation and Sectioning

Following anesthesia with sublethal dose of ether and cooling to 4°C a transcardial perfusion with paraformaldehyde solution (4% in phosphate buffered saline, PBS, Sigma) was performed, the brains were dissected out, and post-fixed for 2-3 days in the same fixative. Then the brains were embedded in agarose, and serial coronal sections (50 μm thick) were cut with a vibration microtome (Intracel, Shepreth Royston Herts, United Kingdom).

### Immunoperoxidase Method

After an overnight washing in phosphate buffer, the sections were pre-treated with 3% H_2_O_2_ for 5 min, and then with 20% normal horse serum for 1.5 h, to suppress the background staining. These, and the following steps all included a rinse in phosphate buffer, interposed between the changes of reagents. The anti-GFAP reagent (polyclonal rabbit antiserum, DAKO, Galstrup, Denmark, Code Nr Z0334, RRID AB10013382) was diluted to 1:200 (28 ng/ml antibody concentration) in phosphate buffer containing 0.5% Triton X-100. In this solution the sections were incubated for 40 h at 4°C. Biotinylated anti-rabbit and streptavidin-biotinylated horseradish peroxidase complex (for the specifications of chemicals see Appendix 2) were applied subsequently, each in a dilution of 1:100, for 1.5 h at room temperature. The immunocomplex was visualized by diaminobenzidine reaction, 0.05% 3,3′-diaminobenzidine in 0.05 M Tris-HCl buffer (pH 7.4) containing 0.01% H_2_O_2_, for 10 min, at room temperature. No structure-bound diaminobenzidine reaction product was found, when the primary antibodies were omitted. Rat brain sections were used for positive controls. The sections were mounted, dried in air, covered with DePeX, and coverslipped.

Photomicrographs were taken by a DP50 digital camera mounted on an Olympus BX-51microscope (both from Olympus Optical Co., Ltd., Tokyo, Japan). Digital images were processed using Photoshop 9.2 software (Adobe Systems, Mountain View, CA, United States) with minimal adjustments for brightness and contrast.

### Pre-embedding Electron Microscopical Immunohistochemistry

In this case 0.5% glutaraldehyde was added to the perfusion solution for a better fixation. Immunoperoxidase method was carried out as above except for that Triton X-100 detergent was reduced to 0.1% to decrease the tissue destruction. Following the immunoreaction, the sections were immersed for 30 min into a 1% osmium tetroxide solution in phosphate buffer (0.1 M, pH 7.4) then rinsed in phosphate buffer, and dehydrated through an upgrade ethanol series up to absolute ethanol and then propylene oxide, and finally embedded into epoxy resin (Durcupan, Fluka). Semithin, and then ultrathin sections were cut with a Reichert Ultracut S ultra-microtome. The photomicrographs were taken by a JEOL 100B electron microscope equipped with a Sys Morada digital camera.

### Identification of Brain Details

It followed the descriptions of Ariens-Kappers et al. (1960), Nieuwenhuys (1997a, b), Nieuwenhuys and Meek (1997). In the case of some species, sketches of mapping studies were also taken into consideration: López et al. (2013, senegal bichir, *Polypterus senegalus*), Rupp and Northcutt (1998, white sturgeon, *Acipenser transmontanus*), Parent and Northcutt (1982, spotted gar, *Lepisosteus oculatus*), Medina et al. (1994, European eel, *Anguilla anguilla*); Singru et al. (2008, walking catfish, *Clarias batrachus*), Arevalo et al. (1992; tench, *Tinca tinca*), and Parent et al. (1978, sunfish, *Lepomis gibbosus*).

## Results

### Telencephalon

The difference between the eversive telencephalon of Actinopterygii, and the evaginative telencephalon found in the sarcopterygian-tetrapod clad is shown in Figure 2. In brief, telencephalic parts positioned dorsomedially in tetrapods turn over, and reach a ventrolateral position. The hemispheres are solid, and enclose no lateral ventricles, but a T-shaped common forebrain ventricle separates them, and surrounds their dorsolateral surfaces. The roof of the ventricle is an epithelial lamina, which corresponds to the roof plate of the original neural tube. This thin layer cannot be preserved in Vibratome sections, therefore, it is not seen in the photomicrographs.

**FIGURE 3:**
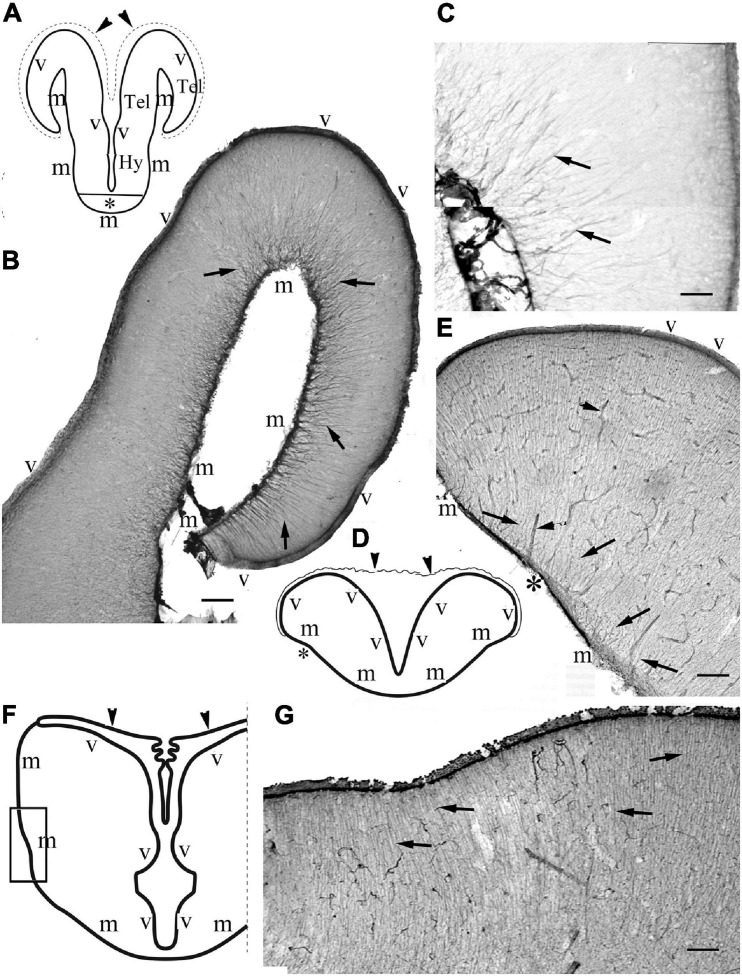
Non-teleost telencephala: the radial glial processes do not converge fan-shaped. **(A)** Sketch of bichir prosencephalon redrawn after López et al. (2013); m,v: meningeal and ventricular surfaces, Hy, Tel: hypothalamus, telencephalon; arrowheads and dotted line: the epithelial roof of ventricle and the choroid plexus (in the photomicrographs it is torn, not visible); asterisk: optic chiasma. **(B)** Bichir telencephalon, m,v: meningeal and ventricular surfaces. Arrows: radial glia. Scale bar:100 μm. **(C)** Bichir telencephalon, enlarged detail. Arrows: radial glia. The processes are almost parallel, do not converge. They cannot be followed to the ventricular surface Scale bar: 40 μm. **(D)** Sketch of sterlet telencephalon redrawn after Nieuwenhuys (1997b), with the position of panel **(E)**, see asterisk; m,v: meningeal and ventricular surfaces; arrowheads: the epithelial roof of ventricle, and the choroid plexus. **(E)** Sterlet telencephalon. m,v: meningeal and ventricular surfaces. Arrows: radial glia, the thick lines (arrowheads) are vessels, asterisk is positioned as in panel **(D)**. The meningeal ends of the radial processes show some convergence but do not gather to a common center. Scale bar: 100 μm. **(F)** Sketch of gar telencephalon redrawn after Parent and Northcutt (1982), with the position of panel **(G)**; m,v: meningeal and ventricular surfaces; arrowheads: the epithelial roof of ventricle, and the choroid plexus. **(G)** Lateral detail of a gar telencephalon. The panel has been turned to right, for the correct position see panel **(F)**. Arrows: end-parts of glial processes arriving to the meningeal surface parallel to each other. Scale bar:100 μm.

The telencephala of the non-teleost groups are shown in Figure 3. In bichir the telencephalon is a thin lamina, which curves lateral- and downward, so the originally most dorsal pallial division comes to lie far lateral (Figures 3A,B), and a deep groove separates the medial and the everted lateral parts. The lengths of the ventricular and meningeal surfaces are almost equal. The glial processes were radial, almost parallel with each other, did not converge (Figures 3B,C). In sterlet (Figures 3D,E) the pallium is thicker than in bichir, the eversion is moderate, the meningeal surface only shortened slightly as compared to the ventricular one. The meningeal ends of the radial processes only converged moderately; they did not gather to a common center. In gar (Figures 3F,G) the eversion is also moderate, a mild sulcus is only found on the outer, lateral surface of telencephalon. The glial processes only curved gently, and showed no or very weak convergence; they were almost parallel with each other.

**FIGURE 4:**
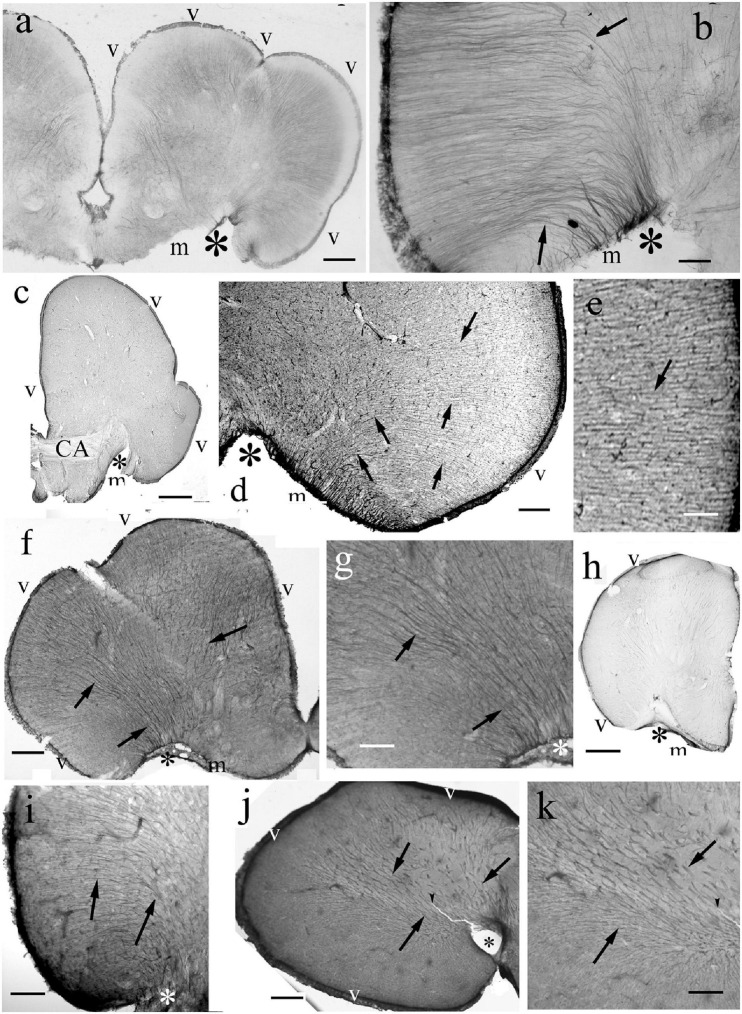
Teleost telencephala: ‘fan-shaped’ convergence of the radial glial processes. Asterisk: the center of eversion, arrows: radial glia, note the intense curvature, m,v: meningeal and ventricular surfaces. **(a)** Eel telencephalon, the eversion is complete, asterisk marks its center. Scale bar: 120 μm. **(b)** Detail of an eel telencephalon. The radial processes converge to the center of eversion (asterisk), the entorhinal fissure. Note the intense curve of processes (arrows) on the lateral part. Scale bar: 50 μm. **(c)** Silhouette of a section of a knifefish telencephalic hemisphere, CA: ant. commissure. Scale bar: 700 μm. **(d)** Detail of an knifefish telencephalon near the center of eversion. Scale bar: 120 μm. **(e)** An enlarged detail of the lateral ventricular surface seen in panel **(d)** for a better demonstration of the glial processes. Scale bar: 40 μm. **(f)** Goldfish telencephalon. Scale bar: 250 μm. **(g)** Lateral part of goldfish telencephalon enlarged. Arrows and asterisks mark identical details here and in the previous panel. Scale bar: 120 μm. **(h)** Silhouette of a section of a catfish telencephalic hemisphere. Scale bar: 500 μm. **(i)** Detail of a catfish telencephalon. Asterisks mark identical details here and in the previous panel. Scale bar: 120 μm. **(j)** Sander telencephalon. Scale bar: 200 μm. **(k)** Midpart of sander telencephalon enlarged. Scale bar: 100 μm.

The ‘typical’ eversion is found in teleosts, see first the eel (Figures 4a,b). It resulted in a fan-shaped re-arrangement of radial glia. They originated from the large and convex dorsal (ventricular) surface, and converged on the shorter and concave basal (meningeal) surface to the center (the pivot point) of eversion near the entorhinal fissure. The curvature of processes was more intense on the lateral side of telencephalon (Figure 4b). The processes gradually thickened toward the meningeal surface. In all the other teleosts studied the astroglial system was similar to this (Figures 4c–k).

**FIGURE 5:**
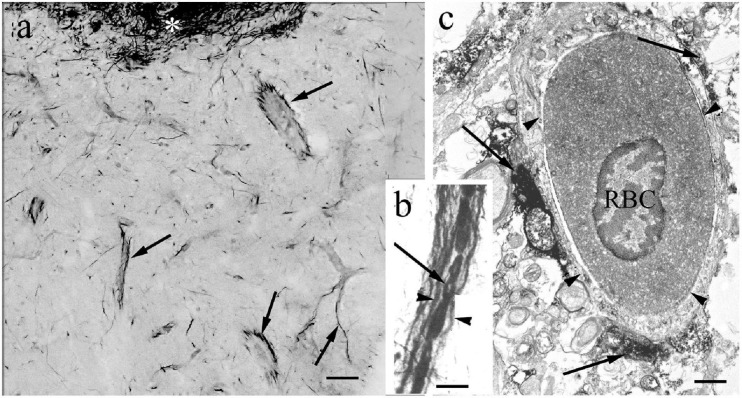
The perivascular glia. In contrast to the other Gnathostomata, in Actinopterygii the perivascular glial processes are not perpendicular to the vessels but course along them. **(a)** Glial processes (arrows) around vessels (goldfish). Asterisk: periventricular glial plexus. Scale bar: 40 μm. **(b)** Glial processes (arrowheads) along a vessel, between them red blood cells. Arrow points to the site of a nucleus devoid of the non-specific peroxidase reaction of hemoglobin (carp). Scale bar: 10 μm. **(c)** Electron microscopic cross-section of a vessel (arrowheads). RBC: a red blood cell with nucleus. Arrows: glial processes. (goldfish). Scale bar: 1 μm.

In contrast to the other Gnathostomata, in Actinopterygii the perivascular astroglial processes were not perpendicular to the vessels but coursed along them (Figures 5a–c).

### Diencephalon and Mesencephalon

**FIGURE 6:**
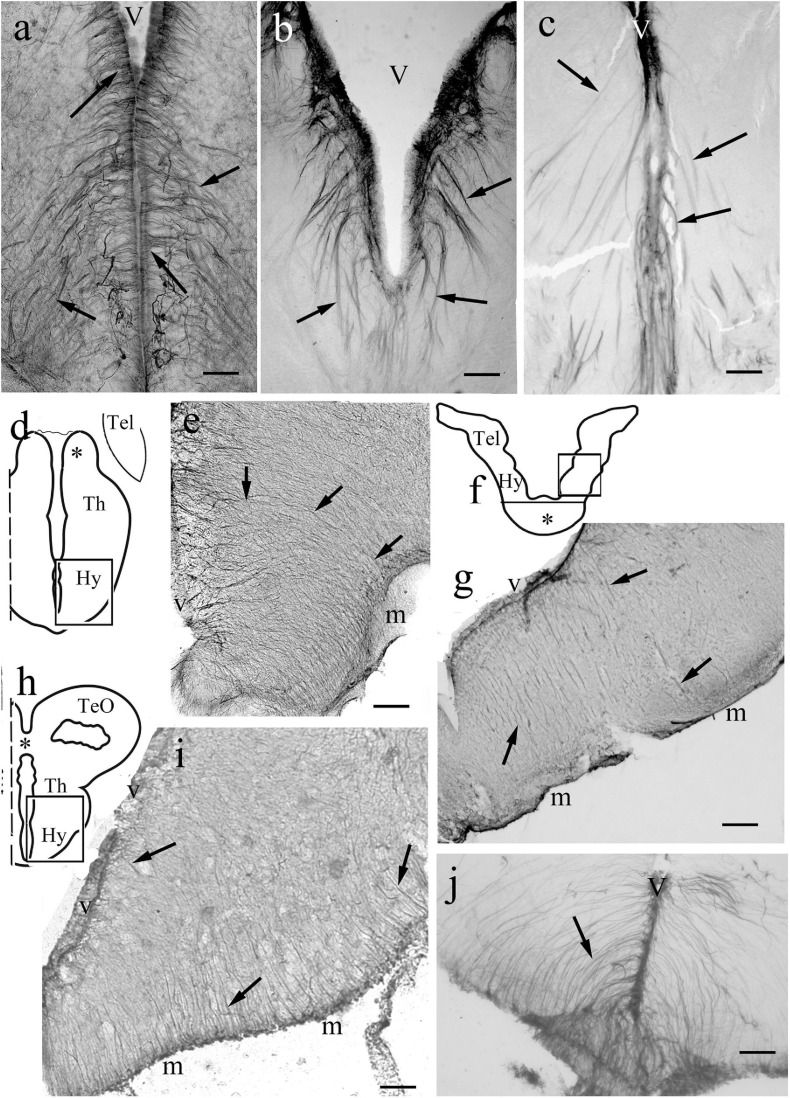
Diencephalon. **(a–c)** The diencephalic ventricle (V) in eel, carp, and sander. Note the oblique thick processes (arrows). In eel and carp the ependymal origin is recognizable well. Scale bars: 200 μm. **(d)** Sketch of bichir diencephalon redrawn after López et al. (2013) with the position of panel **(e)**. Hy, Th: hypothalamus, thalamus, Tel: posterior end of telencephalon; asterisk: habenula, wavy line: the thin roof of ventricle **(e)** Bichir hypothalamus, caudal part, penetrated with arcuate processes (arrows). m, v: meningeal and ventricular surfaces. Scale bar: 100 μm. **(f)** Sketch of sterlet prosencephalon redrawn after Rupp and Northcutt (1998) with the position of panel **(g)**. Hy: hypothalamus, Tel: telencephalon, line and asterisk: optic chiasm. The roof of the ventricle is not shown. **(g)** Sterlet hypothalamus, m, v: meningeal and ventricular surfaces, arrows: radial glia. Scale bar: 100 μm. **(h)** Sketch of gar diencephalon at the posterior commissure (asterisk) redrawn after Parent and Northcutt (1982), with the position of panel **(i)**. Hy, Th: hypothalamus, thalamus. TeO: optic tectum. **(i)** Gar hypothalamus. m,v: meningeal and ventricular surfaces, arrows: astroglial processes. Scale bar: 100 μm. **(j)** Eel, median eminence. Arrow: arched glial processes. Scale bar: 80 μm.

The thalamus had a fine radial glia in non-teleosts. In teleosts the astroglial processes were rather thick, and oriented obliquely downward (Figures 6a–c). The habenulae were traversed with straight glial processes (not shown).

**FIGURE 7:**
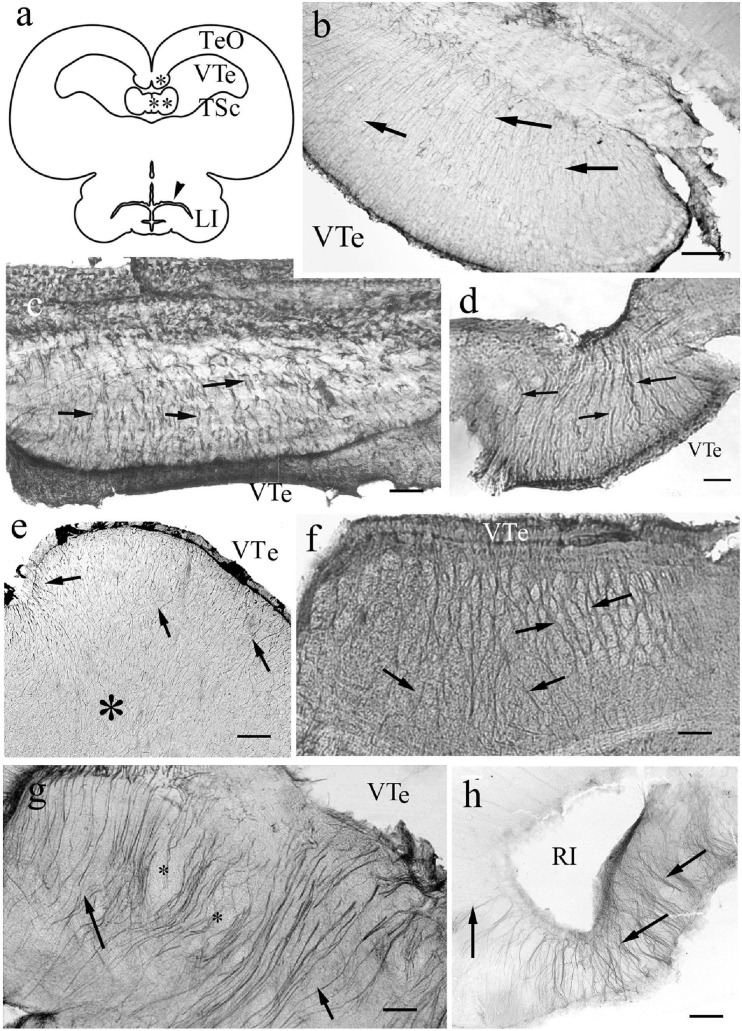
Mesencephalon. **(a)** Sketch of sunfish mesencephalon, redrawn after Parent et al. (1978). LI: lobus inferior; TSc: torus semicircularis; TeO: optic tectum; VTe: tectal ventricle; asterisk: torus longitudinalis; double asterisk: valvula cerebelli, arrowhead: inferior recess of the ventricle. **(b)** Torus longitudinalis, knifefish, VTe: tectal ventricle, arrows: segments of radial processes. Scale bar: 120 μm. **(c)** Torus longitudinalis, carp, VTe: tectal ventricle, arrows: glial processes. Scale bar: 80 μm. **(d)** Torus longitudinalis, catfish, VTe: tectal ventricle, arrows: glial processes. Scale bar: 80 μm. **(e)** Torus semicircularis, bichir. Fine and dense radial processes (arrows), their course is hardly followed inside the tissue (asterisk). VTe: tectal ventricle. Scale bar: 120 μm. **(f)** Torus semicircularis, gar. Light spots (myelinated axon bundles) between radial glial processes (arrows). VTe: surface facing the tectal ventricle. Scale bar: 120 μm. **(g)** Torus semicircularis, eel. Its inner part is rather poor of immunostaining. The neural tracts are surrounded by glial processes. VTe: tectal ventricle, arrows: glial processes, asterisks: lighter spots, myelinated axon bundles. Scale bar: 80 μm. **(h)** Sander, lobus inferior, RI: the recessus inferior of the mesencephalic ventricle, note the radial processes around (arrows). Scale bar: 80 μm.

In the hypothalamus the process systems were fine and radial (Figures 6d–i) in all species studied. The median eminences were similarly penetrated by arched radial processes, which started very thin from the ventricle (Figure 6j). A general view of mesencephalon is provided in a sketch to help orientation (Figure 7a). The torus longitudinalis a paired band, which starts from the posterior commissure, and arches along the medial edges of the two domes of the optic tectum. This structure is not found in bichir (Nieuwenhuys, 1997a). In sterlet it was not preserved in our specimens. In knifefish it contained fine processes (Figure 7b). In more advanced species coarse and slightly contorted ventrodorsal processes formed its astroglial system (Figures 7c,d).

The torus semicircularis emerges from the floor of the tectal ventricle as a slightly curved transverse ridge. In bichir (Figure 7e) and sterlet it contained fine processes, but in the other species it had thicker ones (Figures 7f,g). Its interior was rather poor in GFAP-expressing structures, since only a few glial processes penetrated it separating light spots: myelinated axon bundles. In the lobus inferior (Figure 7h) the process system was radially arranged in every species.

### Optic Tectum

**FIGURE 8:**
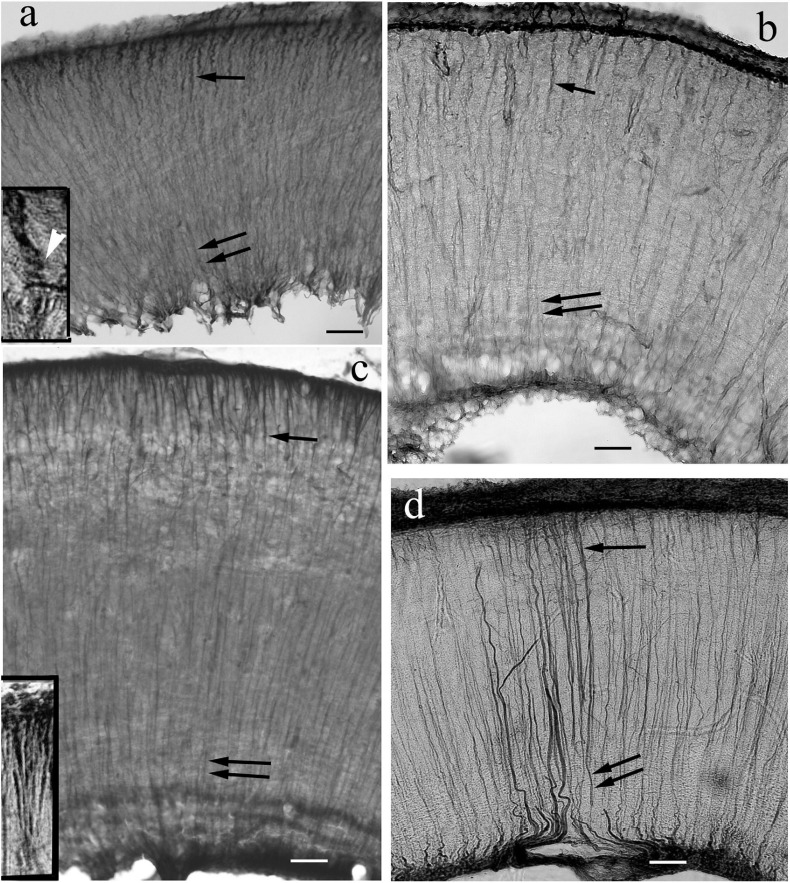
Tectum details of non-teleosts and basal teleosts: processes are immunostained in full length. The meningeal surfaces are upward. The radial processes are visible both near the surface (arrows) and near the ventricle (double arrows). **(a)** Bichir. Its wall is thin related to the other species investigated. Toward the meningeal surface the glial processes thicken and get a little spiralized (see inset, white arrowhead points to the coils). Scale bar: 40 μm, for the inset 15 μm. **(b)** Sterlet. The processes thicken toward the meningeal surface. Scale bar: 40 μm. **(c)** Gar. Comb-like branching of processes (see enlarged in the inset) below the meningeal surface. Scale bar: 40 μm, for the inset: 15 μm. **(d)** Eel. A thin submeningeal zone is distinguished by fine, dense branches. Scale bar: 40 μm.

Regarding its length, it worth’s a separate subchapter. It forms a pair of dome-like hemispheres above a tectal ventricle. Its layers (see e.g., Meek, 1983) cannot be recognized on the basis of GFAP immunostaining. In non-teleosts and basal teleosts (Elopomorpha and Osteoglossomorpha) (Figure 8) the radial glial processes were visualized intensely with immunostaining of GFAP in the full thickness of the tectal wall. In the superficial (submeningeal) zone the processes thickened (e.g., in bichir and sterlet Figures 8a,b), or even formed comb-like branching, e.g., in gar (Figure 8c) and butterfly fish. In the middle and deep (periventricular) zones no branches were visible on the radial processes.

**FIGURE 9:**
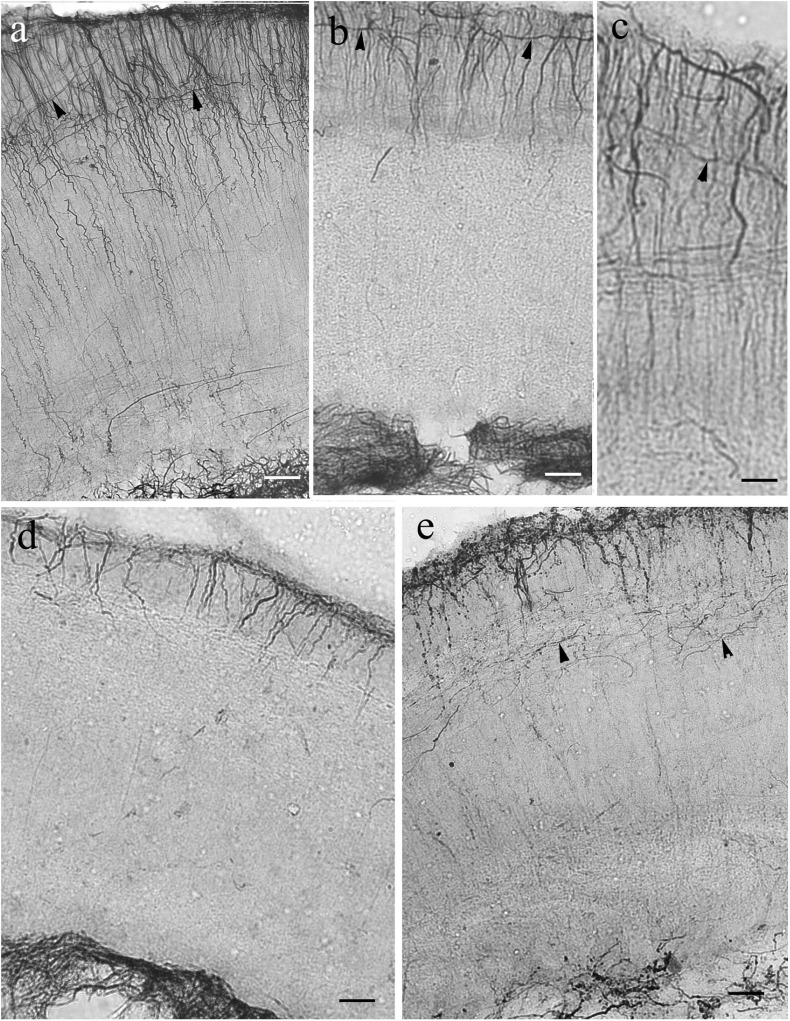
Tectum details of Ostariophysi: processes are not immunostained in full length. The meningeal surfaces are upward. The glial processes (arrows) are visualized well only in the upper part of the section. **(a)** Carp. The upper zone is rich in secondary fibers, among them even horizontal ones. (arrowheads). In the deeper zone glial processes are visible only scarcely. Scale bar: 60 μm. **(b)** bream. The pattern is similar to that of carp; in the deeper zone no processes are visible. Scale bar: 60 μm. **(c)** Bream. The enlarged detail reveals better the rich superficial glial system. Scale bar: 20 μm. **(d)** Bleak. Only the submeningeal part of glial processes is visualized. Scale bar: 60 μm. **(e)** Catfish. The process system is less dense than in cyprinids. Scale bar: 60 μm.

In Ostariophysi (Figure 9) the processes were only visible weakly (Figures 9a,b) or not at all (Figures 9d,e) in the middle zone, and were never seen in the periventricular zone. Except for catfish, in the submeningeal zone the process system was dense with side-branches among them horizontal ones (Figures 9a–c). Catfish had a conspicuously less dense submeningeal process system (Figure 9e).

**FIGURE 10:**
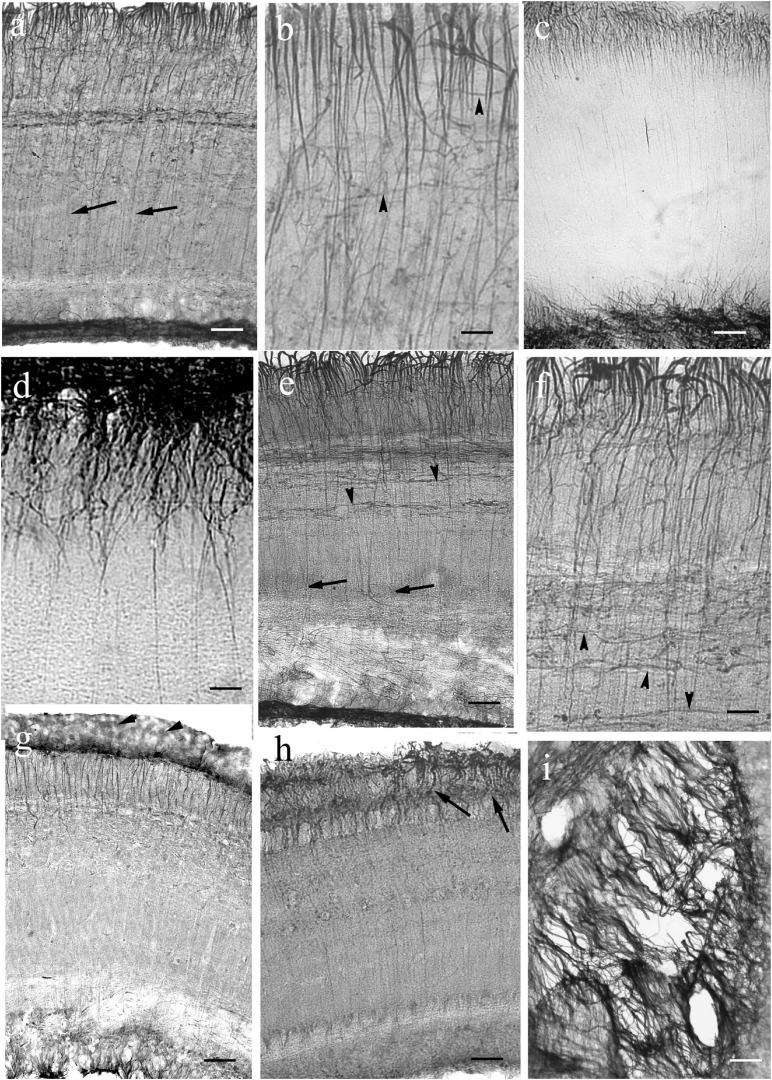
Tectum details of Euteleostei: processes are not immunostained in full length. The meningeal surfaces are upward. The glial processes are visualized intensely only in the upper part of the section. **(a)** Trout. The glial processes although can be followed in the midpart of the section (arrows), but getting thinner, and disappear below. In the upper zone the processes are thick, and branching comb-like. Scale bar: 60 μm. **(b)** Trout. Higher magnification reveals many thin secondary processes among them. Arrowheads: horizontal processes. Scale bar: 20 μm. **(c)** Sunfish. Note the very dense submeningeal process system. Scale bar: 80 μm. **(d)** Sunfish. The submeningeal system enlarged. Scale bar: 20 μm. **(e)** Perch. The glial processes can be followed in the midpart of the section (arrows), but getting thinner, and disappear below; arrowheads: horizontal processes. Scale bar: 60 μm. **(f)** Perch. The submeningeal system enlarged, arrowheads point to horizontal processes. Scale bar: 20 μm. **(g)** Sander. Arrowheads: meningeal end-feet. Scale bar: 80 μm. **(h)** Angelfish. The processes (arrows) thicken toward the meningeal surface, but the density of process system is not so conspicuous. Scale bar: 80 μm. **(i)** The almost ‘bidimensional’ network of glial processes, which lines the tectum, and interconnects its two domes. Pike, tangential section. Scale bar: 15 μm.

In Euteleostei (Figure 10) the tectal astroglial systems were similar to that of Ostariophysi: already in the middle zone the radial processes were labeled very weakly (Figure 10a) or not at all (Figures 10c,e,g,h). Rich submeningeal networks were frequently seen (Figures 10b,d,f). In some sections the meningeal end-feet of processes were in view, see Figure 10g.

Inside the tectum was lined with an almost ‘bidimensional’ network of thick astroglial processes, which connected the two domes. It is demonstrated in a tangential section (Figure 10i).

### Cerebellum

Its main part (corpus) emerges above the rostral medulla; and it is covered by a cortex with molecular and granular layers as in every vertebrate (Meek, 1992). Its size varies from a small ridge to a prominent structure (Ariens-Kappers et al., 1960; Nieuwenhuys, 1967). Its rostral part, the valvula extends below the tectum, as it was shown in Figure 7a.

**FIGURE 11:**
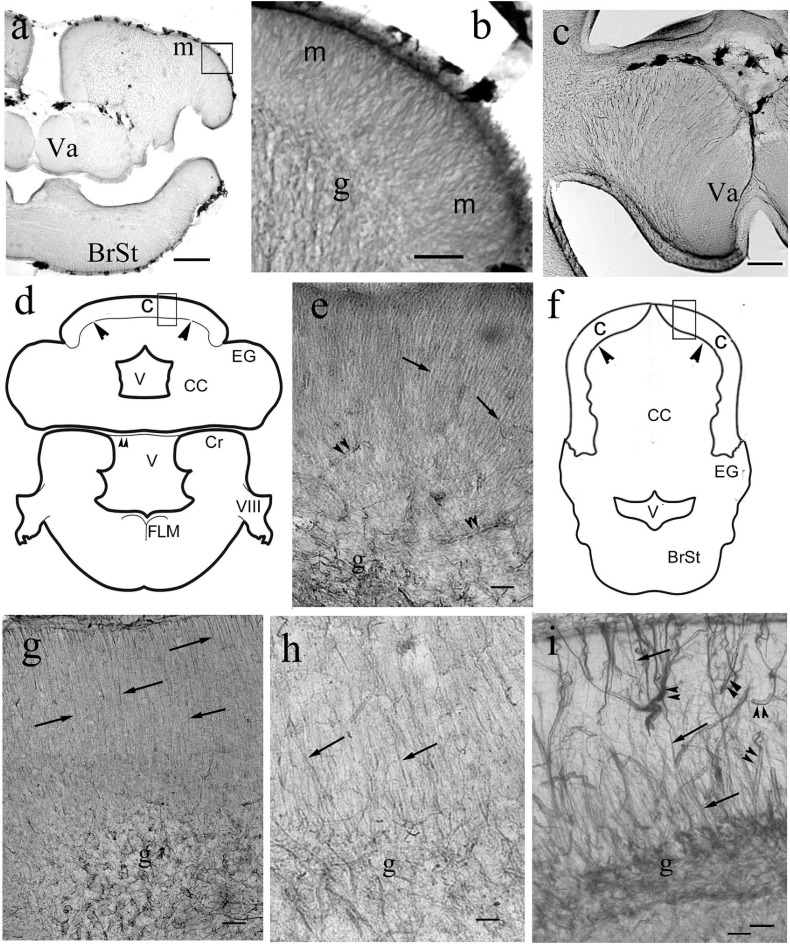
Bergmann-like glia in the cerebella of non-teleosts and basal teleosts. **(a)** Silhouette of bichir cerebellum with the underlying brain stem (BrSt) for orientation of panels **(b,c)**. Va: valvula cerebelli, a part of cerebellum crammed into the ventricle. Scale bar: 300 μm. **(b)** A detail of bichir cerebellum, for its position see panel **(a)**; m: molecular layer with dense process system perpendicular to the surface; g: granular layer. Scale bar: 80 μm. **(c)** Astroglial system of the bicirh valvula cerebelli (Va). Scale bar: 100 μm. **(d)** Sketch of gar cerebellum with the underlying brain stem redrawn after Parent and Northcutt (1982), with the position of panel **(e)**. C and arrowheads: cortex, CC: corpus cerebelli, Cr: crista cerebelli, EG: eminentia granularis, FLM: medial longitudinal fascicle, V: ventricle, VIII: 8th cranial nerve. The sketch of the brain stem also helps orientation in the microphotographs of Figure 13. **(e)** Detail of gar cerebellar cortex. Arrows: Bergmann-like processes, double arrowheads: vessels, g: granular layer. Scale bar: 80 μm. **(f)** Sketch of trout cerebellum for orientation on teleost cerebella, redrawn after Nieuwenhuys and Meek (1997). BrSt: brain stem, C: cortex, CC: corpus cerebelli; EG: eminentia granularis; V: rhombencephalic ventricle; arrowheads: the border of cortex. **(g)** Knifefish. Arrows: Bergmann-like processes, g: granular layer Scale bar: 100 μm. **(h)** Butterfly fish. Marks as in panel **(g)**. Scale bar: 80 μm. **(i)** Eel. Arrows: Bergmann-like processes, double arrowheads: vessels, g: granular layer. Scale bar: 80 μm.

**FIGURE 12:**
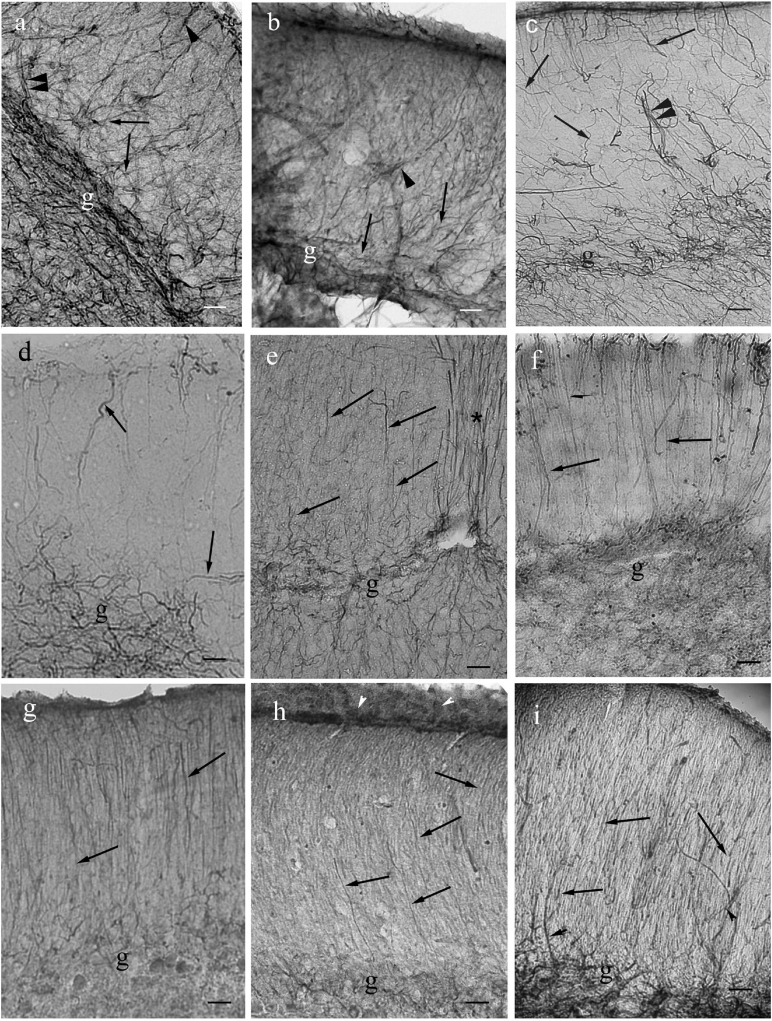
Cerebella in Ostariophysi and Euteleostei. For the positions of the areas see the sketch in the previous figure (panel Figure11f). The meningeal surfaces are upward. Arrows: glial processes; g: granular layer. The coarse (arrowheads), sometimes duplicated (double arrowheads) GFAP-immunopositive lines are most probably vessels (see in carp, Kálmán, 1998). No Bergmann-like process system is recognizable in Cypriniformes (panels **a–d**). If fine processes are observed (arrows) they are not parallel with each other. In catfish (panel **e**) and Euteleostei (panels **f–i**) Bergmann-like processes (arrows) are recognizable. **(a)** Goldfish. Scale bar: 100 μm. **(b)** Bream. Scale bar: 100 μm. **(c)** Carp. Scale bar: 100 μm. **(d)** Bleak. Scale bar: 100 μm. **(e)** Catfish. Asterisk: the thick process population in the midline. Scale bars: 80 μm. **(f)** Trout. Scale bar: 80 μm. **(g)** Perch. Scale bar: 80 μm. **(h)** Sander. Arrowheads: meningeal glial end-feet. Scale bar: 80 μm. **(i)** Lemon cichlid. Scale bar: 60 μm.

In most species the molecular layer contained fine astroglial processes oriented toward the surface like the Bergman glia in mammals (Figures 11, 12), except for Cypriniformes (Figures 12a–d), in which no Bergman-like pattern was observed, although the staining of astroglia in irregular processes proved the successful immunohistochemical reaction against GFAP. In catfish, a representative of Siluriformes, another order of Ostariophysi, however, Bergman-like processes were found (Figure 12e). In this latter panel the thick midline population of processes is also visible; actually, it was also found in the other species. The granular layer had a relatively narrow astroglial plexus (Figures 11, 12).

### Medulla

**FIGURE 13:**
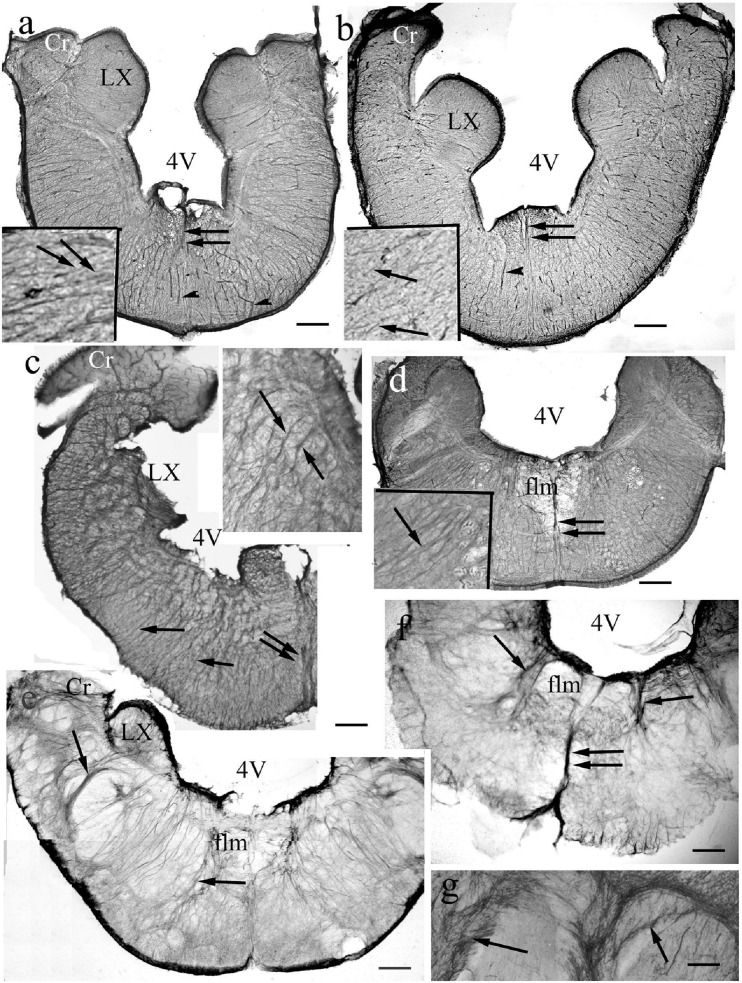
Medulla. 4V: 4th ventricle, its thin roof has not been preserved during sectioning, Cr: crista cerebelli, flm: medial longitudinal fascicle, LX: vagal lobe, arrows: glial processes. double arrows: thick midline glial bundle, arrowheads: vessels. (For orientation also see Figure 11d). **(a)** Bichir. Dense, rather regular radial process system. Scale bar: 120 μm, for the inset 40 μm. **(b)** Sterlet. Dense, rather regular radial process system. Scale bar: 150 μm, for the inset 50 μm. **(c)** Gar. The radial processes are going to be pushed away by myelinated axon groups (light spots). The vagal lobe is damaged. Scale bar: 150 μm, for the inset 50 μm. **(d)** Butterfly fish. The radial processes are going to be pushed away by myelinated axon groups. Scale bar: 200 μm, for the inset 50 μm. **(e)** Eel. The radial system is less regular, the processes separate (arrows) myelinated axon bundles. Scale bar: 200 μm. **(f)** Beam. The radial system is less regular, the processes separate (arrows) myelinated axon bundles. Scale bar: 200 μm. **(g)** A detail of a carp brain stem. Thick bundles of myelinated axons surrounded with glial processes (arrows). Scale bar: 100 μm.

In the medullae (Figure 13) the astroglial systems were rather loose, and evenly distributed in the representatives of non-teleost groups (Figures 13a–c). In teleosts the astroglial processes usually were thick and/or formed bundles, which separated neural tracts incompletely (Figures 13d–g). The midlines were marked by thick dorsoventral bundles of processes. The roofs of the rhombencephalic ventricles, thin epithelial laminae, were not preserved in Vibratome sections.

### Lobus Nervi Vagi

**FIGURE 14:**
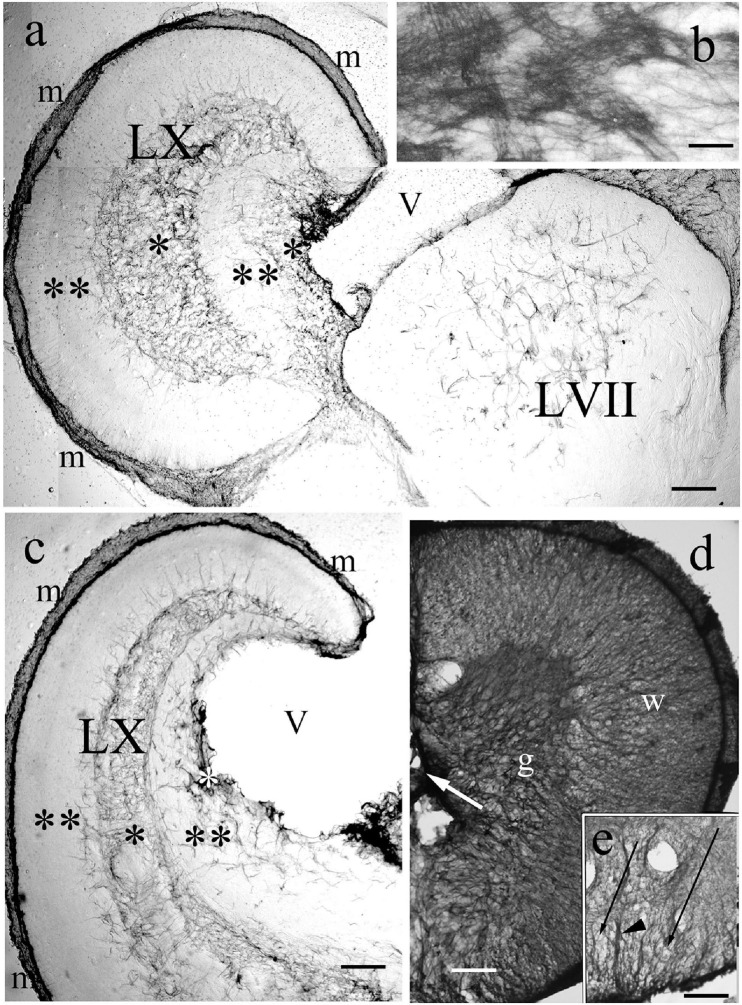
Vagal and facial lobes, and spinal cord. **(a)** Through the facial lobe (LVII); LX: vagal lobe, m: meningeal surface, V: ventricle, asterisks: GFAP-rich layers, double asterisks: GFAP-poor layers. Crucian carp. Scale bar: 300 μm. **(b)** The structure of the GFAP-immunopositive layer of panel ‘a’ enlarged Scale bar: 40 μm. **(c)** Rostral to the level of panel ‘a,’ marks as before. Crucian carp. Scale bar: 300 μm. **(d)** Bichir spinal cord, g: gray matter, dense plexus of glial processes; w: white matter, a less dense system of radial processes. Scale bar: 80 μm. **(e)** The ventral part of the spinal cord of goldfish, arrowhead points to the midline septim, long thin arrows: light spots corresponding to myelinated axon bundles. Scale bar: 80 μm.

The medullae of some Cyprinids has bulky structures, enlarged parts belonging to the 7th, 9th, and 10th cranial nerves, called as facial, glossopharyngeal, and vagal lobes (Figures 14a–c). The last one flanks the rhomboid fossa whereas the first one emerges in its center inside the rhombencephalic ventricle.

The enlargement of these structures accompanied with a characteristic distribution of GFAP-immunopositive elements. In the vagal lobe sections immunostained against GFAP five zones were easy to distinguish: a thin marginal (meningeal) and two thick zones, intermediate and periventricular, which were intensely immunopositive, and two immunonegative zones between them (Figures 14a–c). The periphery of the facial lobe was rather poor in GFAP, while the core was occupied by a plexus of astroglial processes (Figure 14a). The thick GFAP-immunopositive zones contain very dense astroglial plexuses (Figure 14b).

### The Spinal Cord

They were similar in all the species studied. Around the central canal a plexus of astroglial processes was seen corresponding to the position of the gray matter (Figure 14d). This plexus extended coarse glial process bundles toward the surface. The white matter had a similar arrangement to that found around the tracts of the brain stem (Figures 14d,e). The dorsal and ventral glial septa were thick.

## Discussion

### General Consideration

Table 1 summarizes the results and the most important supporting citations. In general, the diversity of astroglial architecture reflexes only moderately the evolutionary diversity of Actinopterygii brains demonstrated, e.g., by Nieuwenhuys and Meek (1997). No variability similar to that of Squamata (Lőrincz and Kálmán, 2020) or Chondrichthyes (Ari and Kálmán, 2008) was found. The predominant GFAP-immunopositive elements were tanycytes, long, tiny fiber-like cells, whereas true astrocytes (stellate-shape cells) were nowhere found.

**TABLE 1:** The summary of results with the most important supporting citations.

	Common features in Actinopterygii	Special features of some groups; #ancestral, ##advanced
Astrocytes^a^	None^1^	
Radial glia or their modifications^b^	Predominate^1^	
GFAP-free areas^a^	None	##Advanced teleosts*: deep tectal zones ##Cyprininae: in the vagal and facial lobes
Telencephalon	Fan-shape modification of radial glia^2^.	#Non-teleosts: no fan-shape modification
Thalamus	Coarse, oblique modification of radial glia^3^.	#Non-teleosts: fine radial glia
Hypothalamus	Radial processes	
Median eminence	Arched modification of radial glia^*c,4*^	
Tectum	Radial glia; GFAP only in their superficial segments^5^	#Non-teleosts and basal teleosts**: GFAP in the full length of processes^d^
Torus longitudinalis^e^	Coarse glial processes^6^	#Non-teleosts and basal teleosts**: fine processes
Torus semicircularis	Coarse radial glia	#Cladistia and Chondrostei: fine processes
Lobus inferior^e^	Radial glia^7^	
Bergmann-like glia	GFAP-immunopositive^f,8^	##Cyprinidae: GFAP-immunonegative^9^,
Medulla	Radial glia^10^	
Vagal and facial lobes^e^	No specific astroglial structure	##Barbinae^11^ and Cyprininae: a characteristic GFAP distribution; GFAP-free areas

*Ostariophysi and Euteleostei. **Elopomorpha and Osteoglossomorpha. Comparison to amniotes and chondrichthyans: ^*a*^Characteristic of batoids, birds and mammals (Horstmann, 1954; Kálmán, 2002; Ari and Kálmán, 2008). ^*b*^Characteristic of sharks and reptiles (Horstmann, 1954; Kálmán, 2002; Lőrincz and Kálmán, 2020). ^*c*^Similarly in reptiles and birds (Kálmán et al., 1993, 1994; Kálmán and Pritz, 2001; Lőrincz and Kálmán, 2020). ^*d*^Similar glial structures in sharks, reptiles and birds, but not in batoids and mammals (Kálmán et al., 1993, 1994; Kálmán and Pritz, 2001; Ari and Kálmán, 2008; Lőrincz and Kálmán, 2020). ^*e*^Found only in Actinopterygii (Ariens-Kappers et al., 1960; Nieuwenhuys and Meek, 1997). ^*f*^Similarly in chondrichthyans (Ari and Kálmán, 2008); and amniotes (Kálmán et al., 1994; Kálmán and Pritz, 2001; Lőrincz and Kálmán, 2020) except for birds (Roeling and Feirabend, 1988; Kálmán et al., 1993). Similar data, in several cases in different species and by different methods (see also Appendix 1). ^1^For reviews see Cuoghi and Mola (2009); Appel (2013)^2^Nieuwenhuys (1962, 1982), Forlano et al. (2001)^3^Onteniente et al. (1983); Ma (1993), Forlano et al. (2001); Arochena et al. (2004)^4^Ma (1993). ^5^Levine (1989); Nona et al. (1989); but silver impregnation proves the continuity of processes to the ventricle (Vanegas et al., 1974; Meek, 1983; Ma, 1993). ^6^Arochena et al. (2004). ^7^Ma (1993). ^8^Dahl et al. (1985); Zupanc et al. (2012). ^9^Dahl et al. (1985); Lucchi et al. (1998). ^10^Onteniente et al. (1983), Rubio et al. (1992); Ma (1993), Forlano et al. (2001); Alunni et al. (2005). ^11^Rubio et al. (1992).

In actinopterygians, no nuclei could be identified on the basis of their GFAP-immunostaining in contrast to that found in mammals (Kálmán and Hajós, 1989; Zilles et al., 1991) and birds (Kálmán et al., 1993). That the light spots coming to sight from the darker background are bundles of myelinated axons, it was discussed earlier (Kálmán, 1998) as well as that the double GFAP-immunopositive lines correspond to perivascular glia. This unique arrangement of astroglial elements along the vessels in fish brains was already described by Achúcarro (1915) by impregnation method. The electron microscopy proves that the staining, which delineates the vessels, is not resulted by a non-specific reaction.

### Tanycytes, Radial Glia, Ependymoglia

The ependymal origin of tanycytes (‘ependymoglia’) was frequently visible, and both radial and non-radial courses were seen. The radial glia seem to be the primary system, and the other astroglial elements (perivascular glia, processes surrounding or following nerve tracts) may be their derivatives (Marcus and Easter, 1995; Kálmán, 1998; Arochena et al., 2004).

The abundant fine protrusions, which were described following Golgi-impregnations (Vanegas et al., 1974; Stevenson and Yoon, 1982; Lara et al., 1989), were not visible in GFAP-stained specimens. This fact is in accordance with the findings of Connor and Berkowitz (1985) who revealed by electronmicroscopic immunocytochemistry that the finest branches of the astroglial arborization do not contain enough GFAP to be detected under light microscope. In several regions Ma (1993) detected NADPH-diaphorase and Forlano et al. (2001) aromatase in radial glial processes.

The course of the glial processes was modified by the morphogenetic processes in some areas, e.g., by the eversion in the telencephalon. A torsion effect of the morphogenetic process also affects the radial course in the inverse telencephala of tetrapods (e.g., in the dorsal ventricular ridge, Kálmán et al., 1994).

### Astrocytes

Astrocytes (i.e., stellate-shaped extraependymal cells) were not found, despite that our former thorough study on carp (Kálmán, 1998) revealed in the medulla a few elements resembling astrocytes. Previous studies based on GFAP-immunostaining have also found a lack of astrocytes in actinopterygian brains (Onteniente et al., 1983; Cardone and Roots, 1990; Rubio et al., 1992; Bernardos and Raymond, 2006; Grupp et al., 2010). Alunni et al. (2005, trout) described round elements without processes as ‘astrocytes.’ A special ‘reticular glia’ containing keratin instead of GFAP was described by Maggs and Scholes (1986, 1990) in the optic nerve of a cichlid.

On the other hand, some studies reported astrocytes applying either classical impregnations, or electron microscopic techniques (King, 1966; Kruger and Maxwell, 1967; Pouwels, 1978; Castejon and Caraballo, 1980; Sensharma and Sensharma, 1981; Lara et al., 1989), although Friede et al. (1969) found no astrocytes in the bowfin (*Amia calva*, Holostei). Note that not all the astrocytes express GFAP (Linser, 1985). Bodega et al. (1993) were unable to demonstrate astrocytes in GFAP-immunostained spinal cord, but found them following impregnations according to Cajal and Rio-Hortega.

These ‘astrocytes,’ however, were similar at two points: (i) they displayed a poor arborization, and (ii) they never predominated a territory but remained scarce. Both features are conflicting to the characteristics of both mammalian and avian astrocytes. Electron microscopic studies revealed the paucity of glial filaments in these cells (Kruger and Maxwell, 1967; Lara et al., 1989), and these authors supposed that they could be incompletely specialized cells, e.g., transient forms to oligodendrocytes.

Reviewing the teleost astroglia Cuoghi and Mola (2009) only mention a very scarce occurrence of true astrocytes (mainly in the optic nerve) rather the occurrence of so-called ‘radial astrocytes’ described first by Sasaki and Mannen (1981, in bullfrog) i.e., multipolar extraependymal cells with a long radial main process. A recent review (Appel, 2013) also summarizes that astrocytes are missing or at least extremely infrequent in Actinopterygii.

### Telencephalon

The forebrains of ray-finned fishes show an increasing complexity from cladistians through chondrosteans and holosteans to teleosts (Northcutt, 2008; Nieuwenhuys, 2009). The bichir pallium is relatively simple; most of perikarya are located in a periventricular layer. In teleosts the pallium can be divided into seven or more subdivisions, compared to the two subdivisions in bichir, and the three or four subdivisions in sterlet (Nieuwenhuys, 1982, 2009; Northcutt, 2008).

The eversion is attributed to that head size constrains the development of forebrain (Striedter and Northcutt, 2006). On the other hand, Costagli et al. (2002) raised the role of the different distribution of reelin in evaginated and everted brains.

The eversion progresses during evolution. In the consideration of Nieuwenhuys (2009) in older groups (Chondrostei, Holostei) the eversion is ‘moderate,’ whereas in most teleosts it is already ‘well marked,’ and ‘most pronounced’ in several percomorphs (e.g., Synbranchiformes among Anabantaria). But the progress is not even: the eversion is only ‘moderate’ in the teleost Salmoniformes, whereas it is ‘most pronounced’ in Cladistia, and the basal teleost Osteoglossomorpha. The holostean bowfin (*Amia calva*) displays a ‘pronounced’ eversion (Nieuwenhuys, 2009); this species in some taxonomies classified as the single representative of Halecomorphi, the sister-group of Teleostei (see Taxonomy subchapter of Discussion). This species, however, was not available for this study.

Fan-shape convergence of radial glial processes due to eversion was earlier mentioned by Nieuwenhuys (1962) on the basis of classical impregnation studies. Applying GFAP immunohistochemistry it was first described in carp (Kálmán, 1998). A strong convergence was described in a batrachoid fish by Forlano et al. (2001, with aromatase immunohistochemistry). The course of radial glia changes in parallel with the eversion. In bichir, sterlet, and gar the ‘fan-shape’ convergence is not formed. Note that in Cladistia the intense cell proliferation and brain thickening, which appear in Chondrostei and Holostei, and in progressed form in Teleostei, do not take place (Nieuwenhuys, 1982, 2009; Northcutt, 2008). Therefore, the eversion of Cladistia although spectacular, and seems to be ‘more advanced’ (Nieuwenhuys, 2009), even represents a basal stage (or a side-branch of evolution, Holmes and Northcutt, 2003, see *their* Figure 1), in which the glial processes do not converge. The ‘fan-shape’ rearrangement of the radial glial processes is formed in teleosts where the eversion is combined with shortening of the meningeal surface.

### Diencephalon and Mesencephalon

Only few study extended over the diencephalon and the mesencephalic tegmentum. These observations were similar to those described in the present study.

The oblique, ventrolateral course of diencephalic and mesencephalic glial processes was described by Onteniente et al. (1983); Ma (1993), Forlano et al. (2001); Arochena et al. (2004). Onteniente et al. (1983) emphasized the unusual thickness of these processes; one can suppose that this thickness indicate transport functions. Ma (1993) supported this supposal filling horseradish peroxidase intraventricularly in sunfish, and reviewed mammalian data on possible transport function of radial glia.

In the torus longitudinalis Arochena et al. (2004), in the lobus inferior Forlano et al. (2001), in the torus semicircularis they and Ma (1993) described the astroglial process systems, which were similar to that seen in the present study.

### Optic Tectum

Its layer system usually consists of six layers in teleosts (Meek, 1983; Nieuwenhuys and Meek, 1997), but it varies in some fishes, e.g., Cerda-Reverter et al. (2008) distinguished only 4 layers in see bass. Kishida (1979) distinguished 8 groups of actinopterygians according to the thickness of layers. The stratum opticum and fibrosum griseum superficiale are thick in species of with good visual perception. In catfish these layers were found to be reduced (Schroeder and Vanegas, 1977). The relatively less dense system of glial processes found in catfish in our study may correlate with this.

Bartheld and Meyer (1987) distinguished 5 types based on the distribution of retinal axons in the layers: I. Non-teleosts; II. Osteoglossoidea and Notopteroidea (basal teleosts; together: Osteoglossomorpha at Betancur-R et al., 2013, 2017); III. Cypriniformes and Characiformes; IV. Siluroidea and Gymnotoidea (both groups III and IV belong to Ostariophysi); V. Neoteleostei; In our study each group had representatives. In the species of groups I and II we found that the radial processes were immunostained in full length to the ventricular surface, whereas in the representatives of groups III to V it was found only in their superficial segments.

In goldfish Levine (1989); Nona et al. (1989) have already found that the tectal glial processes do not contain detectable GFAP immunoreactivity in their full length to the ventricular surface. It is in accordance with the electron microscopic observation of Stevenson and Yoon (1982) that this part is poor in filaments. Vanegas et al. (1974); Meek (1983), however, demonstrated with impregnation methods that the tectal glial processes stretch from the ventricular side to the meningeal surface. Ma (1993) got similar results applying NADPH-diaphorase histochemistry. Our investigation has extended Levine’s observation over all the teleosts, except for their basal groups Elopomorpha and Osteoglossomorpha.

### Cerebellum

The basic cerebellar neuronal architecture is similar in every vertebrates (Meek, 1992). In fish cerebella several authors described Bergman glia (Castejon and Caraballo, 1980, catfish; Somogyi et al., 1990, trout; Meek and Nieuwenhuys, 1991, mormyrid) although not with GFAP-immunohistochemistry. Zupanc et al. (2012) reported GFAP-immunopositive radial glia in the cerebellum of a knifefish. Dahl et al. (1985) found GFAP-immunopositive Bergmann-like glia in representatives of Acanthopterygii. In the gray mullet Arochena et al. (2004) found GFAP-immunopositive elements only in the granular layer but not in the molecular one.

Concerning cyprinids Dahl et al. (1985) found that in goldfish in the molecular layer the glial processes were “less numerous.” Lucchi et al. (1998) obtained similar observation, but Cardone and Roots (1990) found vimentin- and GFAP-immunopositive Bergmann glia in goldfish. Tomizawa et al. (2000) found long perpendicular processes in the molecular layer in the zebrafish but applying C-4 antibody. Therefore Bergmann-like glia most probably exist in Cyprinids, only contain either no GFAP, or with lower or different immunoreactivity Since non-teleosts, basal teleosts have GFAP-immunopositive Bergmann glia, in cyprinids it seems to be an advanced feature but of a separate evolution because siluriforms and euteleosts have GFAP-immunopositive Bergmann-glia.

It is to be noted that mormyrid kneefishes, (Osteoglossiformes, Mormyridae), which have the largest and most sophisticated cerebellum (Meek and Nieuwenhuys, 1991; Nieuwenhuys and Meek, 1997), were not available for us.

### The Medulla Including the Vagal and Facial Lobes

The medullae are quite similar in different actinopterygians (Nieuwenhuys, 1974, 1997a,b, for a recent review: Nieuwenhuys, 2011b), which is reflected by their similar astroglial systems. Our results are in accordance with former descriptions (Onteniente et al., 1983; Rubio et al., 1992; Bodega et al., 1993; Ma, 1993; Forlano et al., 2001; Alunni et al., 2005).

There is one exception: the enlarged facial and vagal lobes, which were found in carp, goldfish and crucian carp in this study. They had a characteristic distribution of GFAP immunopositivity. Similar distributions were found formerly in carp (Kálmán, 1993, 1998) goldfish (Kálmán and Ajtai, 2000), and barb (Rubio et al., 1992). The GFAP-rich zones of the vagal lobe correspond to the “névroglie basale” and “névroglie intérmédiere” described by Achúcarro (1915). Ito (1971) distinguished five zones in the vagal lobe: capsular sensory fibers, sensory neurons, inner sensory fibers (among them the secondary gustatory fibers), motor neurons and ependyma. Of them the first, third and fifth layers correspond to the zones delineated by GFAP immunopositivity, whereas the second and the fourth ones are avoid of GFAP. (Note: the lack of GFAP does not mean the lack of astroglia, see e.g., Linser, 1985). Vimentin does not substitute GFAP in the GFAP-immunonegative zones (Rubio et al., 1992; Kálmán, 1993). Ito (1971), however, described glial cells here in his ultrastructural study. Morita et al. (1983) distinguished 16 layers; the GFAP-free areas correspond to the layers 3 to 11 and the layer 14 of them.

The large and highly layered vagal and facial lobes seem to be a unique evolutionary acquisition of cyprinids (Ariens-Kappers et al., 1960; Finger, 1988; Nieuwenhuys and Meek, 1997; Nieuwenhuys, 2011b). They were formed by an intense gustatory specialization, mainly by the presence of the so-called palatal organ, to receive the gustatory roots. In Siluriformes (also Ostariophysi) and Gadiformes (Paracanthopterygii) the vagal lobes also enlarged but less complex, non-layered (Ariens-Kappers et al., 1960; Finger, 1988). In catfish no characteristic distribution of GFAP like in carp was found (no representative of Gadiformes was studied).

According to Kortschall et al. (1998) the Cyprinidae family has sub-groups according to their lifestyles: Cyprininae and Barbinae (benthic life, ‘chemosensory’ brains), Abraminae (intermediate waters, acoustico-lateralis, visual brains), Alburninae; (surface-dwellers, ‘vision-acustico-lateral’ brains). The above described GFAP distribution was found only in Cyprininae (this study) and Barbinae (Rubio et al., 1992), but not in bream and bleak (representatives of Abraminae and Alburninae).

### Evolutionary Tendencies of Astroglia in Actinopterygii

According to the classification of Butler and Hodos (2005), there are less advanced ‘laminar’ (type I), and more advanced ‘elaborated’ (type II) brains in every main vertebrate clad. The main difference is attributed to that in the ‘elaborated’ brains the neurons migrate to a larger distance than in the ‘laminar’ brains. The brains of Teleostei belong to the ‘elaborate’ type, whereas the non-teleost actinopterygians have ‘laminar’ brains. The difference in the astroglial architectures of non-teleost and teleost telencephala may be a phenomenon of this, as well as the difference in the full-length and incomplete GFAP immunostaining of the glia in the optic tecta of less and more advanced groups.

Within teleosts a comparison of basal groups (e.g., elopomorphs, osseoglossomorphs) with advanced ones (e.g., percomorphs) reveals a shift of brain mass toward higher order integration centers. Telencephalon, optic tectum, inferior lobes of the hypothalamus, and the cerebellum are, therefore, relatively large (Ariens-Kappers et al., 1960; Ridet and Bauchot, 1990), mainly in predator species (Cerda-Reverter et al., 2008).

As it was mentioned above, Kortschall et al. (1998) found that the brain expresses a large variability correlated with the lifestyle (they call it ‘ecomorphology’). The species investigated in our study represented different lifestyles: in muddy deep waters (catfish, carp), near the surface (bleak, butterfly fish), in the intermediate zones (perch, sander, bream), some of them were predators (catfish, sander, trout, eel), others were not. However, despite either the evolutionary changes of brain anatomy, or the different lifestyles, the differences of the astroglial architecture were moderate, except for the vagal lobe in Cyprininae and Barbinae, which was described above. The lack of GFAP immunopositivity in cyprinids (but not in catfish) seems to be as a result of their separate evolution; which factor underlay it, it is not clear.

### Comparison of Astroglia Evolution: Actinopterygii vs. Chondrichthyes and Amniotes

Our former studies on rat (Kálmán and Hajós, 1989; Zilles et al., 1991), chicken (Kálmán et al., 1993), turtle (Kálmán et al., 1994), and caiman (Kálmán and Pritz, 2001) brains suggested that the appearance of GFAP-immunonegative areas as well as that of astrocytes are apomorphic features. Avian homologs of some GFAP-rich turtle or *Caiman* brain areas were frequently almost free of GFAP (e.g., most of DVR, the superficial layers of the tectum, the molecular layer of the cerebellum, Kálmán, 2002, 2009). The results on Squamata species, another florescent and diversive group were in accordance with these tendencies (Lőrincz and Kálmán, 2020).

Astrocytes are also predominant in batoids in the telencephalon and mesencephalon (Horstmann, 1954; Wasowicz et al., 1999; Kálmán and Gould, 2001; Ari and Kálmán, 2008). Most of these areas in batoids are also poor in GFAP (Kálmán and Gould, 2001; Ari and Kálmán, 2008). Note, the Chondrichthyes although appeared earlier than Actinopterygii, yet batoids are not ‘ancestral’ to the latter group but results of a long parallel evolution (Carroll, 1988; Aschliman et al., 2012; Guinot and Cavi, 2016). These comparative data have also been introduced into Table 1.

These tendencies were not found in Actinopterygii, although one can suppose that the GFAP immunonegativity of the evolutionarily ‘new’ layers in the cyprinid vagal lobe, the lack of GFAP immunopositivity in the deeper zones of tectum in advanced teleosts, and in the cyprinid cerebella may allude to them. However, in the cyprinid cerebellum vimentin does not substitute GFAP (carp, Kálmán, 1993) in contrast to the avian cerebellum (chicken, Roeling and Feirabend, 1988; Kálmán et al., 1998). Opposite to the mammalian and avian brains, in fish (goldfish, Kálmán and Ajtai, 2000) lesions were unable to provoke GFAP expression in GFAP-immunonegative areas. This suggests that in Actinopterygii where there is GFAP immunonegativity, it can be attributed to the absence of cells capable of GFAP production rather than its functional repression, in contrast to birds and mammals but like in batoids.

It seems that in Actinopterygii the different astroglial structures developed by the modification of radial glia (see also Kálmán, 1998) rather than by acquiring astrocytes like in amniotes and chondrichthyans. The brain remained relatively small and its wall remained thin. Correlation of forebrains and body weights show that reptile generally possess forebrains sized similarly or two times larger than ray-finned fishes of comparable body size; this proportion for mammals, birds, and batoids can be up to 15 or 20 (Northcutt, 1981). According to the hypothesis of Reichenbach et al. (1987) the evolutionary appearance of astrocytes was induced by the thickening of brain wall.

### Taxonomy

As it was mentioned at Figure 1, the tree of Actinopterygii was adapted from recent studies supported by DNA studies (Betancur-R et al., 2013, 2017). It was later modified by the same group (Hughes et al., 2018); however, these changes did not concern our topic essentially, but should have made Figure 1 too complex, therefore, they have not been introduced into the figure. Especially concerning the Cyprinidae we consulted Mayden et al. (2008). There are, however, divergent opinions. Some taxonomies (e.g., Lauder and Liem, 1983; Carroll, 1988; Nieuwenhuys and Meek, 1997) use two separate taxa instead of Holostei: the group represented by the single species bowfin (*Amia calva*) is ‘Halecomorphi,’ the sister-group of Teleostei (they together: Holostomi), and the rest (i.e., gars and paddlefishes) forms the group Ginglymodi. These authors also mean that Osteriophysi are within Euteleostei; Centrarchidae and Cichlidae are families within Perciformes; and Osteoglossomorpha emerged earlier than Elopomorpha. Inoue et al. (2001) shares this latter opinion; and Northcutt (2008) also separates Ginglymodi and Halecomorphi. On the other hand, Nelson et al. (2016) has similar viewpoints as Betancur-R et al. (2013, 2017). A recent study (Schönhut et al., 2018) separated Leuciscinae from Cyprinidae as a separate family (Leuciscidae). It is to be noted that Lauder and Liem (1983); Carroll (1988) applied paleontologic classifications including fossil taxa, whereas Betancur-R et al. (2013, 2017), Hughes et al. (2018); Schönhut et al. (2018) based on DNA analyses as well as Inoue et al. (2001).

## Conclusion

Despite their high diversity, and the evolutionary alterations, the diversity of the astroglial architecture is moderate in Actinopterygii. The important apomorphic phenomena seen in Chondrichthyes and Amniotes: true astrocytes and extended GFAP-free areas did not arise.

## Data Availability Statement

The datasets presented in this article are not readily available because original photomicrographs are available upon reasonable request at kalmanprof@gmail.com; requests to access the datasets should be directed to MK, kalmanprof@gmail.com.

## Ethics Statement

The animal study was reviewed and approved by Committee on the Care and Use of Laboratory Animals of the Council on Animal Care at the Semmelweis University of Budapest, Hungary (22.1/3491/003/2008).

## Author Contributions

VM, DL, and OS performed the histology. MK contributed to the evaluation and manuscript. All authors contributed to the article and approved the submitted version.

## Conflict of Interest

The authors declare that the research was conducted in the absence of any commercial or financial relationships that could be construed as a potential conflict of interest.

## Appendix 1

### The Specification of Species Mentioned in the Citations

To save place in the text, the names of species described in the papers cited are given here. Taxonomic categories above ordo are only given to help positioning where the ordo is not shown in Figure 1. Categories within ordo are only given where more than one species of the ordo are mentioned in Figure 1 *plus* citations. The scientific names and taxonomic positions are given in their original forms applied by the author cited, but the present forms are also indicated.

Achúcarro (1915)^∗^, Ito (1971)^∗∗^, Kálmán (1993, 1998), Onteniente et al. (1983); Lara et al. (1989)^*+**^ Common carp (*Cyprinus carpio*), Cypriniformes, Cyprinidae, Cyprininae.

Pouwels (1978)^∗^, Somogyi et al. (1990)^∗∗^, Nieuwenhuys and Meek (1997)^∗∗∗^, Alunni et al. (2005). Rainbow trout (*Salmo irideus*, presently: *Oncorhynchus mykiss*), Salmoniformes.

Rubio et al. (1992), Bodega et al. (1993)^*+**+GFAP^ Iberian barb (*Barbus -* presently *Luciobarbus - comiza*), Cypriniformes, Cyprinidae, Barbinae.

Marcus and Easter (1995), Finger (1988)^∗∗∗^, Tomizawa et al. (2000); Bernardos and Raymond (2006) Zebrafish (*Danio rerio*), Cypriniformes, Cyprinidae, Danioninae#.

Stevenson and Yoon (1982)^*+**^, Meek (1983)^∗^, Dahl et al. (1985); Levine (1989), Nona et al. (1989); Cardone and Roots (1990), Lucchi et al. (1998); Kálmán and Ajtai (2000), Grupp et al. (2010), Goldfish (*Carassius auratus*), Cypriniformes, Cyprinidae, Cyprininae.

Nieuwenhuys (1997b)^∗∗∗^, Kálmán and Ari (2002) Sterlet (*Acipenser ruthenus*), Acipenseriformes.

Arevalo et al. (1992)^∗∗∗^ Tench *(Tinca tinca)*, Cypriniformes, Tincinae.

Arochena et al. (2004) Gray mullet (*Chelon labrosus*), Ovelantariae, Mugiliformes,

Castejon and Caraballo (1980)^∗∗∗^ Madamango sea catfish (*Arius -* presently: *Cathorops – spixi*), Siluriformes, Ariidae.

Cerda-Reverter et al. (2008)^∗∗∗^ Sea bass (*Dicentrarchus labrax*), Perciformes, Moronidae.

Cuoghi and Mola (2009) Review of papers on several teleost species.

Dahl et al. (1985) Sea raven (*Hemitripterus americanus*), Percomorpharia, Scorpeniformes@;

Sea bass (*Centrotristes striata*), Perciformes, Serranidae; Scup (*Stenotomus chrysops*), Perciformes, Sparidae; Tautog (*Tautoga onitis*), Percomorpharia, Labriformes.

(and goldfish, see above).

Forlano et al. (2001)^∗∗∗^ Plainfin midshipman (*Porichthys notatus*), Batrachoidiformes, Batrachoidaria.

Friede et al. (1969)^∗∗^ Bowfin (*Amia calva*) Holostei, Amiiformes.

Holmes and Northcutt (2003) Senegal bichir (*Polypterus senegalensis*), Cladistia (or Brachyopterygii).

King (1966)^∗^ Eastern gizzard shad (*Dorosoma cepedianum*), Clupeiformes.

Kruger and Maxwell (1967)^∗∗^ Bluegill (*Lepomis macrochirus)*, Centrarchiformes; barred sand bass (*Paralabrax nebulifer)*, Perciformes, Serranidae.

Kortschall et al. (1998)^∗∗∗^Review on telencephala of several teleost species.

Lara et al. (1989)^*+**^ Bocage’s barb (*Barbus* -presently *Luciobarbus*- *bocagei*): Cypriniformes, Cyprinidae, Barbinae; as well as common carp, see above.

Ma (1993)^∗∗∗^ *Lepomis sp*.: Centrarchiformes.

Maggs and Scholes (1986, 1990) Nile tilapia (*Tilapia –* or *Oreochromis – nilotica*), Cichliformes, Cichlidae.

Medina et al. (1994) European eel (*Anguilla anguilla*) Anguilliformes, Elopomorpha.

Meek (1992)^∗∗∗^ Review on cerebella of several teleost species.

Meek and Nieuwenhuys (1991) ^∗∗∗^ Peter’s elephantnose fish (*Gnathonemus petersi*), Osteoglossiformes, Mormyridae.

Morita et al. (1983)^∗∗∗^ Crucian carp (*Carassius carassius*)*:* Cypriniformes, Cyprinidae, Cyprininae.

Nieuwenhuys (1962, 1982)^∗^ Reviews on different actinopterygians.

Nieuwenhuys (1997a)^∗∗∗^ Reedfish (*Erpetoichthys calabaricus):* Cladistia (also named Brachiopterygii).

Parent and Northcutt (1982)^∗∗∗^ Spotted gar (*Lepisosteus oculatus*), Lepisosteiformes.

Parent et al. (1978) Sunfish (*Lepomis gibbosus*), Centrarchiformes.

Rupp and Northcutt (1998)^∗∗∗^ White sturgeon (*Acipenser transmontanus*), Acipenseriformes.

Sensharma and Sensharma (1981)^∗^ Stripped snakehead (*Channa striata):* Anabantiformes, Anabantaria.

Singru et al. (2008)^∗∗∗^ Walking catfish (*Clarias batrachus*), Siluriformes, Claridae.

Vanegas et al. (1974)^∗^ Striped mojarra (*Eugerres plumieri)*, Perciformes, Gerreidae.

Zupanc et al. (2012)^∗∗∗^ Brown ghost knifefish (*Apteronotus leptorhynchus)*, Osteoglossiformes, Notopteridae.

^∗^ impregnation study, ^∗∗^ electron microscopy, ^∗∗∗^ other study; unlabeled: GFAP study.

@ in Betancur-R et al. (2013): Scorpenidae, within Perciformes. #In some taxonomies they classed to Rasborinae.

## Appendix 2

### Sources of Chemicals

The specification of the primary antiserum (anti-GFAP) is given in the Methods. Other chemicals:

3,3-diaminobenzidine (DAB): Amersham, United Kingdom.

Avidin-biotinylated horseradish peroxidase compl. (ABC): Vector Labs, Burlingam, United Kingdom.

Anti-rabbit immunoglobulin (goat): Vector Labs, Burlingam, United Kingdom.

DePeX: Sigma-Aldrich, St. Luis, MO, United States.

Ether: Reanal, Budapest, Hungary.

Hydrogen peroxide (H_2_O_2_)*_:_* Reanal, Budapest, Hungary.

Normal horse serum: Vector Labs, Burlingam, United Kingdom.

Paraformaldehyde: Merck, Darmstadt, Germany.

Phosphate buffered saline (PBS):Sigma-Aldrich, St. Luis, MO, United States.

Tris–HCl buffer: Sigma-Aldrich, St. Luis, MO, United States.
